# Spontaneous coronary artery dissection: a clinically oriented narrative review

**DOI:** 10.1038/s44325-024-00004-y

**Published:** 2024-05-10

**Authors:** Quan Dang, Sonya Burgess, Peter J. Psaltis, Sarah Fairley, Jacqueline Saw, Sarah Zaman

**Affiliations:** 1 Westmead Applied Research Centre, Faculty of Medicine and Health, University of Sydney, Sydney, Australia; 2 Department of Cardiology, Nepean hospital, Sydney, Australia; 3 University of Sydney, Sydney, Australia; 4 Vascular Research Centre, Lifelong Health Theme, South Australian Health and Medical Research Institute, Adelaide, Australia; 5 Department of Cardiology, Central Adelaide Local Health Network, Adelaide, Australia; 6 Adelaide Medical School, University of Adelaide, Adelaide, Australia; 7 Department of Cardiology, Wellington Hospital, Wellington, New Zealand; 8 Division of Cardiology, Vancouver General Hospital, University of British Columbia, Vancouver, BC Canada; 9 Department of Cardiology, Westmead Hospital, Sydney, Australia

**Keywords:** Interventional cardiology, Medical imaging, Acute coronary syndromes

## Abstract

Spontaneous coronary artery dissection (SCAD) is an important cause of acute coronary syndromes (ACS), with a higher incidence in younger female patients. It is also associated with pregnancy, delivery, and the post-partum period. Despite an exponential rise in the volume of SCAD-focused research and publications within the past decade, SCAD is still a poorly understood condition, with a paucity of randomised controlled trial data. This review discusses the pathophysiology, clinical presentation, diagnosis and management of SCAD alongside areas for future research.

## Introduction

Spontaneous coronary artery dissection (SCAD) is an increasingly recognised cause of acute coronary syndrome (ACS) in younger women. SCAD was first described in the British Medical Journal in 1931, in a 42-year-old woman who developed severe chest pain and died suddenly; with autopsy finding a ruptured dissecting right coronary artery^1^. Since this first case report, research interest into SCAD has increased substantially^2^. SCAD has a strong predilection for young and middle-aged women. While the prevalence of SCAD is less than five percent of all ACS, it accounts for a third of cases in women under the age of 50^3–6^. This paper will discuss contemporary understanding of the pathophysiology, clinical presentation, diagnosis and management of SCAD.

## Definition and classification of SCAD

**Fig. 1:**
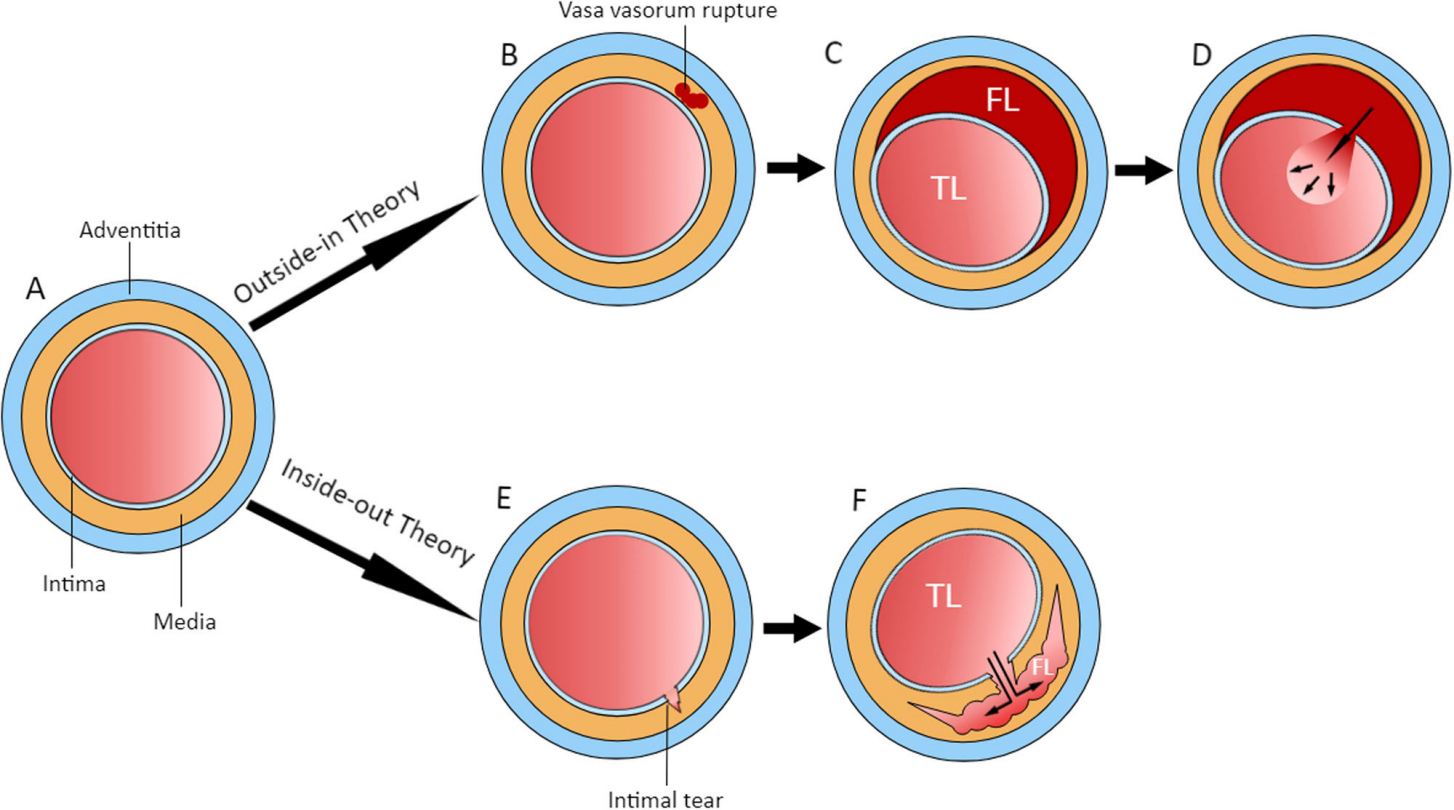
Pathophysiology of SCAD. Schematic representation of the cross-section of a normal coronary artery with three layers **A**. In the Outside-in Theory, a vasa vasorum ruptures in the media, causing a small intramural haematoma **B**. This haematoma may increase in size and compress the true lumen **C** and/or result in a rupture of the intima leading to the haematoma emptying into the true lumen **D**. In the Inside-out Theory, a tear first appears in the intima **E**, followed by sub-intimal dissection caused from blood entering from the lumen creating a false lumen. FL false lumen, TL true lumen. The false lumen may expands as the dissection spread further between layers of the arterial wall **F**.

SCAD is typically characterised by the sudden formation of an intramural haematoma between any of the three layers (intima, media, and adventitia) of the coronary artery wall, forming a false lumen (Fig. 1). It is not related to atherosclerotic, traumatic, or iatrogenic causes^7,8^. An intimal tear or fenestration may form a communication between the intramural haematoma and true lumen of the artery. SCAD may cause abrupt mechanical obstruction to coronary blood flow, resulting in acute myocardial infarction (MI), and less commonly, acute cardiac arrhythmia and sudden cardiac death.

**Fig. 2:**
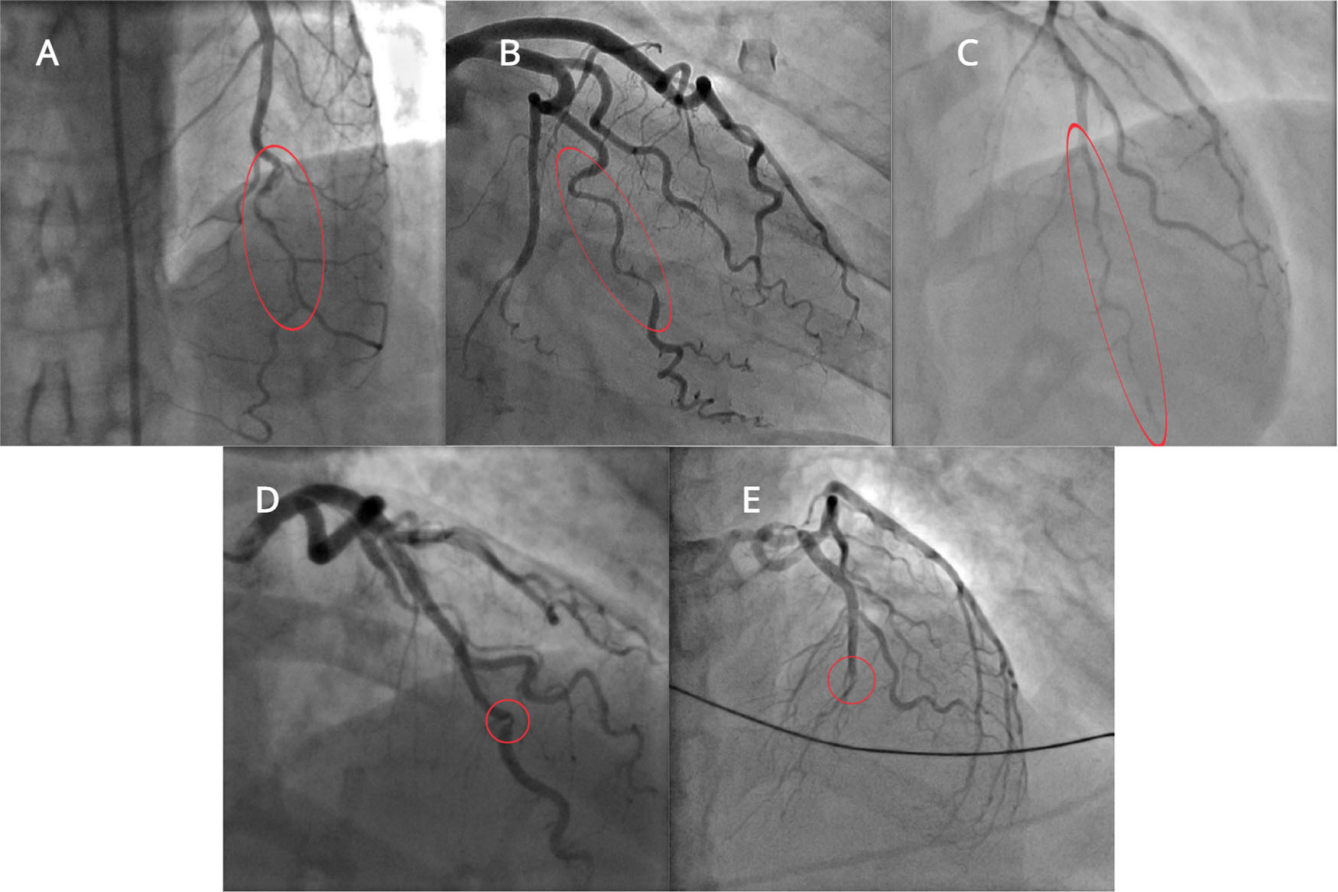
Angiographic types of spontaneous coronary artery dissection. **A** Type 1 SCAD; **B** Type 2 A SCAD; **C** Type 2B SCAD; **D** Type 3 SCAD (confirmed on intracoronary imaging); **E** Type 4 SCAD (completely occluded vessel, confirmed on intracoronary imaging and after repeat angiography showed healing). Images sourced from the ANZ-SCAD Registry^89^. SCAD spontaneous coronary artery dissection.

Invasive coronary angiography (ICA) is the current gold-standard investigation to diagnose SCAD. Saw et al.^9,10^ proposed three types of SCAD based on angiographic appearance (Table 1, Fig. 2). In type 1 SCAD, contrast is visible in both the true and false lumens giving the pathognomonic appearance of multiple lumens on angiography. In type 2 and type 3 SCAD, contrast can be seen in the true lumen only with the appearance of a diffuse (type 2) or short (type 3), often tubular, stenosis. It may be difficult to differentiate type 3 SCAD from an atherosclerotic lesion on coronary angiography alone and intravascular ultrasound (IVUS) or intracoronary optical coherence tomography (OCT) may be required to confirm the diagnosis. Type 2 SCAD can be further sub-classified into type 2a, where the haematoma is sandwiched between normal artery segments proximal and distal to the affected segment, and type 2b, where the SCAD/haematoma extends to the very distal aspect of the vessel. In 2017, Al-Hussani et al.^11^ proposed a type 4 SCAD, where the coronary artery is completely occluded. Type 4 SCAD can be difficult to differentiate from other causes of coronary occlusion and can usually only be diagnosed by additional intracoronary imaging after vessel opening, or subsequent angiography showing full vessel healing^11^. Saw et al.^12^ found ~30% of SCAD affected arteries to be occluded distally however, a long segment of diffuse narrowing prior to the occlusion would usually be seen, meaning these cases would be classified as 2b. A registry study of 1002 SCAD affected arteries found that the prevalence of type 1, type 2, and type 3 SCAD were 29.0%, 60.2%, and 10.8%, respectively^12^.

**Table 1:** Coronary Angiography Classification of SCAD

Type of SCAD	Description
Type 1	Multi-lumen appearance with visible contrast in both true and false lumens
Type 2 Type 2a Type 2b	Appearance of a diffuse stenosis (> 2 cm) with contrast visible in the true lumen only SCAD does not extend to the tip of the vessel SCAD extends to the tip of the vessel
Type 3	Appearance of a short stenosis (< 20 mm). Contrast visible in the true lumen only, may mimic atherosclerotic lesion
Type 4	Total occlusion of the coronary artery. Need to exclude thromboembolism as a cause and subsequent angiography should demonstrate healing of the vessel in keeping with the natural progression of SCAD

*SCAD* Spontaneous coronary artery dissection.

## Epidemiology of SCAD

SCAD was once thought to be a rare condition^13^ however, there has been an increased incidence that likely reflects rising awareness amongst clinicians and improved access to intracoronary imaging^7,14^. In published studies, the proportion of SCAD among patients with ACS ranges from 1 to 5%^15–19^ of which 80–95% of all SCAD cases are found in women^5–29^. In women under 50 years of age with MI, the prevalence of SCAD is up to 35%^3–6^. In one study, SCAD was one of the most common causes of pregnancy-associated acute MI, described in 43% of MI cases^20^. This female predominance appears to be relatively consistent across the world, except for the Persian Gulf area, where only 50% of patients with SCAD, in a single study, were reported to be female^21^. The cause of this different gender proportion is not fully understood. Possible contributing factors might include the inadvertent inclusion of atherosclerosis cases, differences in diagnostic criteria, gender-related differences in diagnostic strategies, or ethnicity-related difference in susceptibility.

In multi-ethnic countries like the United States of America or Canada, Caucasians make up about 90% of SCAD registry patients but comprise no more than 70% of the general population^12,22–24^. This over-representation of Caucasian people may reflect selection bias, or true differences in SCAD susceptibility between ethnicities. While studies in China and Japan provided data about SCAD in Asian populations, there is limited data about the occurrence of SCAD in black or Indigenous people^6,25^. One possible explanation is the earlier development of atherosclerosis in indigenous people which may preclude the diagnosis of SCAD^26,27^.

## Pathophysiology and risk factors for SCAD

There are two main theories about the mechanism of SCAD: the inside-out and outside-in theories (Fig. 1)^7,8^. In the inside-out theory, the initial insult is a tear involving the coronary intimal layer. Blood then dissects to the sub-intimal space to form a haematoma. In the outside-in theory, the initial insult is proposed to be a rupture of the vasa vasorum, leading to a haematoma forming in the arterial wall. This haematoma may then rupture into the true lumen. This is supported by findings on OCT imaging of SCAD cases which showed a lack of communication between the true and false lumen^28–30^. Features suggesting pressurisation in the false lumen includes larger lumen size when indexed to lesion length and absence of contrast in the false lumen even with fenestrations^30^. It is believed that the outside-in theory is the more common mechanism, thus, postulating that bleeding is the primary mechanism rather than dissection/tearing. This observation may change how we manage people with SCAD in the future regarding long term use of antiplatelet therapy.

The observation that SCAD mostly affects young women, and that SCAD is the most common cause of pregnancy-associate MI suggest a role of female hormones in SCAD susceptibility^20^. Oestrogen, a female sex hormone, has been known to also have effects on angiogenesis, vasodilation, and autonomic regulation^31^. These effects may explain its association to SCAD, although the exact mechanism remains unclear.

Structural weakness in the arterial wall is a predisposing risk factor for SCAD. Multiple arteriopathies have been reported in SCAD cases with the most common one being fibromuscular dysplasia (FMD)^12,32–34^. FMD is an arteriopathy affecting small- and medium-sized arteries characterised by disorganised architecture of the arterial wall without an atherosclerotic or inflammatory cause^35,36^. Multiple studies have shown a high prevalence of FMD and other extra-coronary vascular abnormalities (up to 86%) among patients with SCAD^33,37,38^. As a result, screening for FMD and other extra-coronary arteriopathies has been advocated for in all patients with SCAD.

Rare instances of familial cases of SCAD, including in identical twins, have also been reported^39,40^, suggesting genetic susceptibility. SCAD has also been observed in cases of rare inherited vascular diseases, such as Ehlers-Danlos syndrome, Marfan syndrome, and Loey-Dietz syndrome^41–43^. However, this is uncommon and genetic testing targeting rare pathogenic mutations have low yield^7,8^. Low-frequency allele variants were also found be contributed to SCAD, although not pathogenic. In one study, patients with SCAD were found to be more likely to carry rare variants within the fibrillar collagen genes^44^. SCAD has also been found to be associated with multiple common variants with likely complex polygenic interactions^45^. In a genome-wide analysis looking at more common genetic variants, an allele in the PHACTR1 (phosphatase and actin regulator 1) common genetic locus on chromosome 6p24 (rs9349379-A) has been found to be associated with SCAD^46^. Interestingly, this allele is also associated with FMD, which may contribute to the association between these two conditions. A recent genome-wide association meta-analysis has identified 16 risk loci for SCAD and these loci were most enriched in vascular smooth muscle cells and fibroblast^47^.

## Clinical presentation and natural progression of SCAD

As SCAD tends to cause an acute obstruction of coronary blood flow, ACS is the most common presentation. In a large prospective SCAD registry in Canada, the proportion of ST elevation MI, non-ST elevation MI, and unstable angina were 27.9%, 69.9%, and 0.4%, respectively^12^. In the same cohort, chest discomfort was the most common symptom (91.5%) while arrhythmia was uncommon at 1.1%. Sudden cardiac death is a rare occurrence. In a recent autopsy study of people with sudden cardiac death, only 0.3% was found to have SCAD^48^.

Most cases of SCAD heal spontaneously with time. In studies where repeat coronary angiogram was performed following the index SCAD event, healing was reported in the majority of patients at a median of 5 to 39 months^38,49,50^. A recent retrospective study reported the rate of SCAD healing on computed tomography coronary angiography (CTCA) 80 days after the index event to be 71.4%^51^. It should be noted that as repeat coronary angiography would be more likely to be done in patients with recurrent symptoms, the true rate of healing is likely to be higher than the numbers reported in these studies.

## Diagnosis and role of multimodality imaging Invasive coronary angiography (ICA)

Catheter-based ICA is the current gold standard test for the diagnosis of SCAD. Useful features to differentiate between SCAD and atherosclerosis plaque are given in Table 2. In cases of diagnostic uncertainty, administration of intracoronary GTN, use of intravascular imaging (where safe and feasible), CTCA, and cardiac magnetic resonance imaging can be useful. The importance of diagnostic clarity affords appropriate management, which in SCAD is different to the management of atherosclerotic disease.

**Table 2:** Useful characteristics to differentiate between SCAD and atherosclerosis on invasive coronary angiography

Characteristics	SCAD	Atherosclerosis plaque
Lesion appearance	Linear, tubular, contrast staining of double lumen	Irregular
Usual location	Mid to distal	Proximal
Non-culprit coronary arteries	Normal arteries, free of significant stenoses	Multiple stenoses in coronary arteries
Length of lesion	Usually >10 mm	<10 mm
Calcification	Uncommon	Possible
Coronary artery tortuosity	Common	Less common

*SCAD* Spontaneous coronary artery dissection.

## Intracoronary imaging in SCAD

Intracoronary imaging modalities, including IVUS and OCT, are not routinely performed in all cases of SCAD but may be useful in diagnostic uncertainty and to guide management. In one study, OCT confirmed the diagnosis in 11 out of 17 patients with angiographically diagnosed SCAD^52^.

**Fig. 3:**
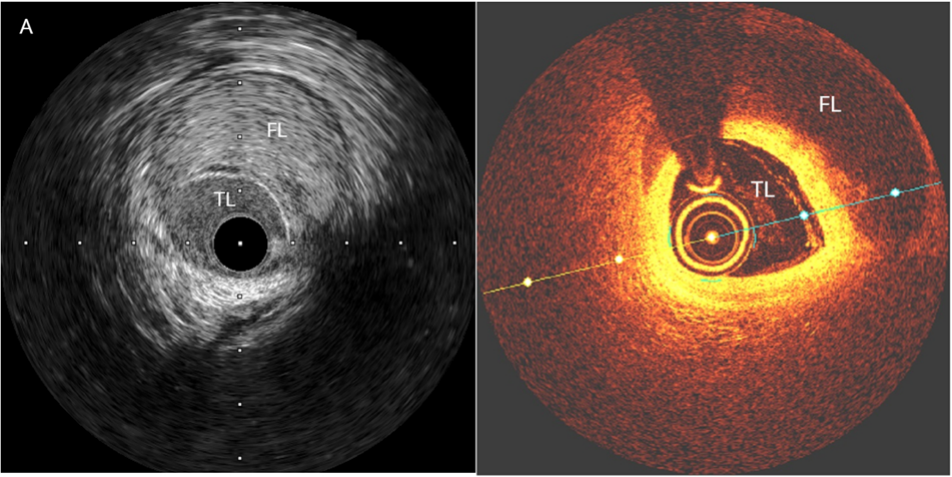
Intracoronary imaging findings of spontaneous coronary artery dissection. **A** intravascular ultrasound image of SCAD; **B** Optical coherence tomography image of SCAD. Images sourced from the ANZ-SCAD Registry^83^. SCAD spontaneous coronary artery dissection, FL false lumen, TL true lumen.

Both imaging modalities, OCT and IVUS, have advantages and disadvantages in the setting of SCAD. OCT has higher spatial resolution, with a greater ability to visualise an intimal tear (Fig. 3). IVUS has better depth penetration and is therefore more useful in larger vessels. IVUS has the additional advantage of not requiring contrast injection during image acquisition as contrast injection risks propagation of the haematoma. In one study of 63 patients with SCAD who underwent OCT, 7.9% had OCT-related complications, but none resulted in a major adverse cardiac event^30^. Another limitation of these intravascular imaging is the size of these devices (approximately 1 mm), limiting their use in small, tortuous, and distal vessels, areas commonly involved in SCAD cases. In the case of diagnostic uncertainty, particularly in larger, more proximal vessels or type III SCAD, either modality can help confirm the diagnosis. When percutaneous intervention is required in cases of SCAD, intracoronary imaging can confirm wire position (luminal or subintimal), guide stent size and length and reduce the risk of stent malapposition following intramural haematoma reabsorption.

## Computed tomography coronary angiography (CTCA) in SCAD

CTCA has the advantage of being a non-invasive diagnostic procedure and can demonstrate the presence of coronary calcification or plaque suggestive of underlying atherosclerosis as the alternative cause of coronary narrowing^53,54^. One report has proposed that CTCA be used as the first diagnostic test in patients with high suspicion for SCAD^55^. In another small case study, CTCA correctly identified SCAD in 14 out of 18 lesions (78%), with most of the missed lesions located in small vessels^53^. This highlights the main limitation of CTCA in SCAD; the lower spatial resolution that limits detection of SCAD in smaller vessels and therefore, prevents CTCA from excluding the diagnosis. Therefore, CTCA as a primary diagnostic strategy is limited and it remains an adjunctive test to ICA in people with suspected SCAD. An emerging role for CTCA in SCAD is in the long-term follow-up where it has been shown to demonstrate large vessel healing. In a recent study, the sensitivity and specificity of CTCA in detecting unhealed SCAD lesions were 72% and 53.8%, respectively^51^. Currently, however, there is no consensus on the optimal use of CTCA as a follow-up modality.

## Acute management

As most cases of SCAD heal spontaneously, where possible, a conservative approach should be used. Percutaneous Intervention carries significant risk due to the higher chance of iatrogenic (catheter-induced) coronary dissection, and wiring, ballooning, or stenting can all promote propagation of the dissection or intramural haematoma^56^. Guidewires and/or stents may inadvertently be placed into a false lumen and stent sizing may be challenging due to the presence of a large intramural haematoma. This can lead to late stent malapposition, restenosis and stent thrombosis. The American Heart Association consensus document has proposed an algorithm to manage acute SCAD^7^. In the Canadian SCAD registry of 750 patients, 86.4% were managed conservatively, with only 2.3% of these patients subsequently requiring invasive treatment. Urgent revascularisation may be required in the case of haemodynamic instability or high-risk anatomy (left main dissection or double proximal vessel disease). In this instance, both percutaneous coronary intervention (PCI) or coronary artery bypass grafting (CABG) can be considered. When PCI is performed, use of intravascular imaging with IVUS or OCT may be helpful, but the risks should also be considered. This enables identification of the coronary wire in the true lumen, correct stent sizing (1:1) and correct stent length to cover the entire dissected segment. There are several options for PCI. The aims of PCI are to restore coronary flow, control the dissection and subsequent haematoma, thus avoiding proximal and distal propagation. Both the proximal and distal edges of the dissection can be stented with subsequent cutting balloon deployment / stent deployment in the mid segment. Alternatively, a single long stent can be used to ensure adequate coverage into normal vessel (at least 5-10 mm beyond proximal and distal borders of the SCAD lesion). Cutting balloons alone can be used to treat SCAD. These are usually deployed at the site of maximal luminal narrowing or as guided by intravascular imaging where possible. In a recent review of 32 cases of SCAD treated with cutting balloon, restoration of flow to Thrombolysis In Myocardial Infarction (TIMI) 3 were achieved in 93.8% of the cases with distal propagation of the haematoma as the most commonly reported complication (18.8%). In the same review, IVUS was used in 53.1% of the cases and provided useful information to guide the intervention^57^. If CABG is performed, then venous grafts may sometimes be the graft of choice, with the knowledge that the affected vessel will likely heal within a few months, and the graft will subsequently occlude. However, there is limited evidence to support decisions surrounding choice of revascularisation strategy in people with SCAD.

## Long-term treatment of SCAD and prevention of recurrence

In one study, the 3-year risk of SCAD extension and recurrence were 3.5% and 2.4%, respectively^58^. There are currently no randomised controlled trial data to guide treatment of SCAD. In 2018, the European Society of Cardiology (ESC) and the American Heart Association (AHA) published their first scientific statements on SCAD, with recommendations on pharmacologic treatments with some variation to treatment of people with atherosclerotic ACS^7,8^. However, the latest 2023 ESC ACS guidelines has the recommendation that patients with SCAD be given the same pharmacologic treatments as other patients with ACS^59^.

The role of antiplatelet therapy in SCAD has been controversial. As SCAD involves the formation of an intramural haematoma, there is a theoretical risk of promoting further dissection/ haematoma with the use of antiplatelets or anticoagulants used in ACS. However, SCAD is also a prothrombotic state due to the potential exposure of blood to the sub-intimal tissue, and reduced coronary flow from true lumen obstruction. Thrombus formation both in the haematoma and the true lumen has been observed^60^. Antiplatelet use may therefore be beneficial in reducing this thrombus burden. In their scientific statements, the AHA and ESC suggested at least a single antiplatelet agent (aspirin), to be used acutely. In a European observational study, MACE at one year occurred in 18.9% with dual antiplatelet use vs 6% with single antiplatelet use in conservatively managed patients (hazard ratio [HR] 2.62, 95% confidence interval [CI] 1.22–5.61, *p* = 0.013)^61^. An observational study on 327 patients with SCAD found that aspirin lowered the risk of SCAD recurrence on univariate analysis (HR 0.36, 95% CI 0.18–0.73, *p* = 0.004) but not on multivariable analysis^62^. In patients treated with PCI, dual antiplatelet therapy according to current guidelines is recommended.

Based on observational registries, the most common practice worldwide has been to treat people with SCAD with dual antiplatelet therapy (usually aspirin and clopidogrel) for 3 months, followed by single antiplatelet therapy (usually aspirin) lifelong^63^. However, even single antiplatelet therapy can create problems in younger female patients, with menorrhagia a frequent adverse event^7,8^. In these cases, individual case-by-case discussion is needed, given the current lack of efficacy data supporting antiplatelet therapy following confirmed SCAD. Currently no randomised trial data is available to guide management. The Beta-blockers and Antiplatelet agents in patients with Spontaneous Coronary Artery Dissection (BA-SCAD) trial is currently enroling and will address this important and unresolved knowledge gap.

The use of anticoagulation in the acute phase carries the same considerations as discussed in the antiplatelets section above. In the acute settings, anticoagulation is usually commenced prior to coronary angiography. In the absence of other indications, it was recommended that anticoagulation be stopped once a diagnosis of SCAD has been made^7,64^.

In a systematic review of n = 4206 patients with SCAD, beta-blockers were found to be significantly associated with a reduced risk of SCAD recurrence after adjustment for confounders; RR of 0.51 (95% CI 0.33 -0.77, *P* = 0.0013)^65^. Beta-blockers have therefore been recommended in all patients with SCAD^7,8^. Once again, side effects of fatigue or reduced exercise intolerance are particularly common in younger patients with SCAD and are often a limiting factor in the ongoing use of beta-blockers.

In a retrospective study (*n* = 87, median 47 months of follow up) statin use was found to be associated with increased SCAD recurrence; 50% vs 8%, *p* = 0.022^66^. However, this was not reproduced in other studies, and a systematic review (*n* = 295) did not find an association between statins and recurrent SCAD^65^. Therefore, while statins are not routinely recommended for people with a confirmed diagnosis of SCAD, they can be used if the diagnosis is unclear, or the patient has hypercholesterolaemia, or atherosclerosis seen on ICA or CTCA. Similarities and differences in management between ACS secondary to SCAD versus atherosclerotic-related ACS is summarised in Table 3.

**Table 3:** Comparison of management between SCAD ACS and atherosclerotic ACS

Treatment	SCAD ACS	Atherosclerotic ACS
Antiplatelets	Single antiplatelet therapy or DAPT for the first 3 months is reasonable. DAPT if stenting	DAPT for 12 months
Statins	No, unless another indication	Yes, Moderate to high dose
Beta-blocker	Yes, Lifelong if tolerated	Yes, Usually at least 12 months
ACEI or ARB	Used for LVEF impairment or hypertension	Yes
Cardiac rehabilitation	Yes	Yes
Screening for FMD	Yes	No

*ACEI* angiotensin converting enzyme inhibitor, *ACS* acute coronary syndrome, *ARB* angiotensin receptor blocker, *DAPT* dual antiplatelet therapy, *FMD* fibromuscular dysplasia, *LVEF* left ventricular ejection fraction; *SCAD* spontaneous coronary artery dissection.

Patients with SCAD-related ACS and left ventricular ejection fraction (LVEF) impairment should be treated with Angiotensin converting enzyme inhibitors (ACEI) or angiotensin receptor antagonists (ARB) in keeping with current guidelines^59^. The use in patients without LVEF impairment has not been well established. As untreated hypertension has been shown to be a risk factor for SCAD recurrence, ACEI or ARB could be considered as an option to treat hypertension^65^.

Post-SCAD chest pain is a common issue affecting more than half of SCAD survivors^7^. This can be broadly divided into (i) acute chest pain, which should be evaluated in a hospital setting with serial ECGs and cardiac enzyme tests, and (ii) sub-acute or chronic chest pain, which could be managed in an out-patient setting. Chronic chest pain post SCAD could be related to ongoing ischaemia due to non-healing of SCAD, microvascular dysfunction, or unknown causes. In a recent study of 17 survivors with chronic chest pain despite confirmed angiographic healing of SCAD, more than 70% were found to have microvascular dysfunction as indicated by abnormal coronary flow reserve or index of microcirculatory resistance^67^. Anti-anginal medications, such as nitrates or calcium channel blockers could be considered to treat chronic post SCAD chest pain. Protocols for the evaluation and management of chest pain post SCAD have been proposed by the American Heart Association and American College of Cardiology^7,64^.

## Pregnancy and SCAD

SCAD is one of the most common causes of MI in pregnant women with the post-partum period the most at-risk period^68^. Pregnancy or post-partum SCAD has a 4% in hospital mortality rate. From published studies, pregnancy-related SCAD (P-SCAD) usually has higher incidences of ST elevation myocardial infarction, reduced left ventricular ejection fraction, involvement of the left main artery or multivessel SCAD^20,68–70^. In the Canadian SCAD registry, peripartum status was found to be an independent predictor of 30-day and 3-year MACE^12,58^. P-SCAD is best managed with a multidisciplinary team approach. In women who are pregnant, breastfeeding or planning a pregnancy, aspirin is safe and can be continued. Most beta-blockers are safe in pregnancy (excluding atenolol)^71^. As most medications have not been meaningfully tested in pregnancy, other commonly used SCAD medications should be used with caution in pregnancy or are not recommended during pregnancy or breastfeeding including P2Y12 inhibitors such as clopidogrel (category B) or ticagrelor (category C), statins (category X), and ACEI/ARBs (category C or D). These all require special consideration in these women.

## FMD screening

The prevalence of FMD among patients with SCAD was variable between studies^33,72^. In an autopsy study on 18 patients with sudden cardiac death from SCAD, none were found to have FMD^48^. This could be due to the rarity of SCAD causing sudden cardiac death (only 0.3% from the same study), or a low prevalence of FMD. On the other hand, the prevalence of FMD has been reported to be as high as 86%^33^. In the balance of current evidence, FMD screening has been advocated for in all patients with SCAD^7,8^. In a recent systematic review and meta-analysis, FMD was found to be associated with an increased risk of SCAD recurrence (RR, 2.02; 95% CI, 1.03–3.94; *P* = 0.0404)^65^. FMD screening also allows the detection of high-risk vascular malformations which may require close monitoring or intervention. Non-selective angiography of the renal and iliac arteries can be performed at the same time as invasive coronary angiography; however, this will not visualise intracranial vessels. Full FMD screening can be performed with head to pelvis CT angiography. Magnetic resonance imaging (MRI) angiography can be used instead of CT, dependent on local availability and individual factors, but has lower spatial resolution than CT. Some experts advocated for using MRI of the cerebral and neck arteries to better define aneurysm and webs. The risks of screening, including those of false positive and false negatives, should be discussed with patients. An international consensus document has been published to guide the management of FMD^36^.

## Cardiac rehabilitation, physical activity and mental health considerations

Strenuous physical activity has been reported to be a trigger of SCAD, likely due to increased shear stress in the coronary artery. This has raised concerns about physical activity in people with SCAD, including during cardiac rehabilitation. However, multiple studies have demonstrated that cardiac rehabilitation in patients with SCAD is both safe and could improve patients’ well-being^73–76^. It is recommended that all patients with SCAD following an ACS should be referred to cardiac rehabilitation^7,8^. Current cardiac rehabilitation programs usually cater for older patients with atherosclerotic MI, and may be less suitable for younger patients with SCAD^74,77,78^. SCAD-specific cardiac rehabilitation programs have been reported with encouraging results and should be used if available^73^.

Low intensity activities, moderate aerobic exercise, interval training and weight training with low resistance and high repetition have been recommended for people with SCAD^79^. Moderate-intensity activities can be guided by symptoms with graded increase in activity levels. High intensity activities with abrupt movements and/or extreme head positions should be avoided; however, given the lack of robust evidence, case-by-case discussion may be considered.

Psychological disorders, including depression, anxiety, and post-traumatic stress disorder, are highly prevalent among patients with SCAD, especially in the first year after presentation^77,80–82^. The limited understanding of SCAD and the uncertainty about its treatment and prevention could precipitate stress among survivors. In a recent study, anxiety was the most common mental health disorder that developed after a SCAD event^77^. It is possible that these disorders are both a risk factor and a consequence of SCAD^77,83^. Mental health screening in patients with SCAD is recommended^7^. Peer-support group is helpful for people with ACS and such groups have been developed online for SCAD survivors, including by SCAD Alliance, Beat SCAD and SCAD Research Incorporated^84–86^.

## Genetic screening

Genetic screening of all people with SCAD is generally low yield and therefore not routinely recommended^7,8^. Genetic testing may, however, be considered in patients with where a genetic basis is more likely, such as recurrent SCAD, multivessel SCAD, extra-coronary vascular abnormalities, a family history of SCAD or of hereditary connective tissue disease (particularly in a 1st degree relative)^64,87^. During clinical assessment, signs of connective tissue disease, such as joint hypermobility and skin hyperextensibility (suggestive of Ehler-Danlos syndrome), arachnodactyly and ectopia lentis (suggestive of Marfan syndrome), should be sought. Echocardiography may also help detect valvular abnormalities suggestive of connective tissue disorders. In patients who genetic screening is deemed appropriate, gene panels developed for aortopathy and connective tissue disorders can be considered^88^. As with all genetic screening, adequate counselling for the patient and their family is essential.

## Current research and future directions

Until 2015, most of the publications on SCAD were case reports. The development of SCAD registries around the world has allowed significant progress in our understanding of this condition^12,61,66,89^. However, there are still difficulties in analysis of representative people with SCAD globally; limited by the lack of a SCAD-specific item number in the World Health Organisation’s international classification of diseases, and most registries coming from Europe and North America^90^. Given the diagnosis is sometimes unclear on coronary angiography, review of angiography with experienced cardiologists as adjudicators in a core laboratory setting is a feature of current SCAD registries(Canadian-SCAD in Canada, SR-SCAD in Spain, the DISCO in France, the G-SCAD in the Gulf countries, and ANZ-SCAD in Australia and New Zealand)^12,17,89,91,92^.

No randomised controlled study (RCT) on SCAD has yet been published. The currently enroling BA-SCAD trial, recruiting patients in Spain with a 2 × 2 factorial design, will be the first^93^. Patients will be randomised 1/1 into groups of beta-blockers vs placebo, and short (1 month) vs long (12 months) antiplatelet therapy. The study will provide important data about the efficacy and safety of beta-blockers and anti-platelet therapy in patients with SCAD but will likely lack sufficient sample size to report on MACE and death.

Photon-counting CT is a new detector technology that allows higher spatial resolution compared to current detector technology. Early studies have demonstrated good diagnostic value of CTCA using photon-counting detector^94,95^. Due to the higher resolution, this technology has the potential to improve the diagnosis of SCAD with further studies required. The role of artificial intelligence (AI) in medicine has been evolving. AI models have been developed to interpret coronary angiograms with modest results^96^. With more data, this technology may help in the diagnosis of SCAD in the future.

## Conclusion

SCAD is an increasingly recognised cause of ACS. The diagnosis of SCAD can be challenging, and a high level of clinical suspicion in the setting of ACS in a young person or pregnancy-associated ACS, should be combined with multimodality imaging, where required. Large registry studies have allowed advancement in our understanding of the presentation and natural history of SCAD, but RCT data are lacking and urgently needed to provide robust, evidence-based management. Current data suggests that most patients with SCAD can be managed conservatively without intervention and that beta-blockers are associated with a lower risk of recurrence. However, there is still much unknown regarding treatment. Randomised trials are in the process of being developed and performed and are expected to provide more definitive data to guide management of SCAD.
